# Supplementary material for: Scene-relative object motion biases depth percepts
Source: Sci Rep. 2022 Nov 2;12:18480. doi: 10.1038/s41598-022-23219-4 (PMC9630409; doi:10.1038/s41598-022-23219-4)
Supplement: Supplementary file 1 — Supplementary Legends. [file 41598_2022_23219_MOESM1_ESM.docx]

**Video 1:** Example stimuli for the binocular condition. Stimuli for two example trials are shown. In the first trial (same trial as illustrated in Figure 3) simulated self-motion is leftward, and the subject needs to make a smooth eye movement to the right to track the fixation point. The target object has a far depth, and a large leftward motion relative to the scene (5 cm/s). In the second trial, simulated self-motion is again leftward and the target object has a far depth, but there is no scene-relative object motion. In this second trial, motion of the target object relative to the fixation point is due to self-motion and the object’s depth. For correct viewing, the video should be viewed through red-green anaglyphic glasses, with the red filter placed over the left eye.
